# Trialling Locally Made, Low-Cost Bits to Improve Bit-Related Welfare Problems in Cart Horses: Findings from a Study in Senegal

**DOI:** 10.3390/ani13010002

**Published:** 2022-12-20

**Authors:** Mactar Seck, Ruth Jobling, Ashleigh F. Brown

**Affiliations:** 1 Brooke West Africa, Dakar 22482, Senegal; 2 Brooke UK, London EC3A 2BJ, UK

**Keywords:** working equid, equid welfare, lorinery, harnessing, animal welfare, human–animal interaction

## Abstract

**Simple Summary:**

The majority of the bits used for working horses in Senegal are made of construction iron, recovered from the rubble of destroyed houses or near construction sites. The poor design, shape, fit and quality lead to injuries in the horses’ mouths which can impair their welfare by potentially causing discomfort, pain, distress and difficulty eating. To address this, Brooke—a charity aiming to improve the welfare of working horses, donkeys and mules—devised a pilot project to manufacture and trial improved bits for cart horses working in transportation of people and goods. This study has demonstrated the feasibility of producing an alternative bit design for cart horses that is low-cost and locally crafted from aluminium, which can improve upon bit-related aspects of cart horse welfare over a relatively short time period of 21 weeks. The improved bit designs have scope to positively impact upon large numbers of working horses in Senegal and beyond, whilst also representing a potential livelihood or income diversification opportunity for the local artisans who manufacture them and the vendors who sell them.

**Abstract:**

Bits used for cart horses in Senegal are typically made of recovered construction iron and often have defects related to design, shape, fit and metal quality. Consequently, there is widespread presence of bit-related oral injury amongst these equids. It was hypothesised that improving bit design would ameliorate bit-related welfare issues for working cart horses. This study aimed to develop locally made alternative bit prototypes and test their efficacy as less harmful to working horses, and their acceptability to their drivers. Eight animal-based welfare indicators (four physical and four behavioural) were designed to measure positive or negative effects of the new bits. Following a testing phase to appraise and mitigate potential animal welfare risk associated with the alternative bit designs, a total of 540 driver/horse combinations were opportunistically selected across five municipalities in Senegal. Welfare indicators were observed when new bits were introduced and again after 21 weeks of daily use. The results indicated statistically significant improvements in all welfare indicators measured (i.e., lesions on lip commissures, tongue, buccal mucosa and bars; and open mouth, tongue loll, head toss/shake, and head tilt/turn behaviours). None of the drivers reported any difficulty with horse control, nor chose to revert back to their original bits. Whilst acknowledging the limitation of inability to control all potential confounding variables, these preliminary findings suggest the bit itself as an important contributor to oral injury, and the possibility to improve this through alternative bit design that is low-cost, locally produced and acceptable to drivers.

## 1. Introduction

The global population of working equids is estimated as 100–112 million [1], and these animals are engaged in provision of essential domestic, commercial and livelihood support to many millions of people around the world. Working equids support families, communities and national economies, providing invaluable contributions through direct and indirect income-generating activities, social and domestic support, subsistence and food security [2]. Their impact is particularly important in low-income country contexts where mechanised transportation is not widely affordable, and in remote and rural locations where equids often provide one of the few means of transportation for goods and people in hard-to-reach locations. Despite the valuable contributions they make, working equids suffer a range of welfare deficiencies, which are multi-factorial in origin. Working equid welfare may be impaired on account of the physical demands upon them to undertake their work; harsh and dangerous working environments; negative human–animal interaction, including violence during driving in some cases; lack of access to high-quality animal health services; and poor knowledge about husbandry, health and behavioural requirements for equine species. They, and the communities of people reliant upon them, may also be vulnerable to extraneous geographical or political challenges, such as drought, famine, natural disasters or conflict, for which working equids also provide valuable support to community resilience [3].

Brooke is an international charity, founded in 1934, striving to improve the welfare of working horses, donkeys and mules in South Asia, the Middle East, Latin America and sub-Saharan Africa. Brooke began working in Senegal in 2010, initially through partnership, and formally established Brooke West Africa in 2012. The population of working equids in Senegal is estimated as 539,309 horses and 458,693 donkeys [4], and these animals undertake commercial work types including cart transportation of goods and local people in urban and peri-urban locations, and domestic work types including water transportation in rural areas, making invaluable economic and domestic contributions [5]. Brooke West Africa aims to alleviate the problems working equids encounter and improve their welfare mainly through engagement with relevant communities, building capacity of service providers (e.g., veterinarians, farriers, harness-makers) and advocacy for better inclusion of working equids in livestock sector development policies.

**Figure 1:**
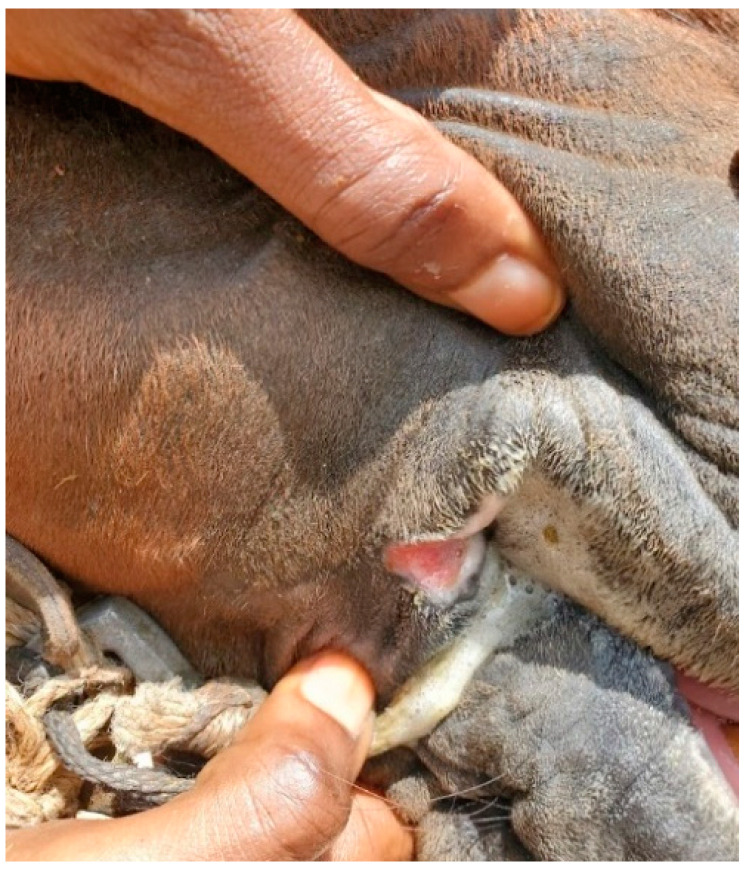
Bit-related lip lesion on a cart horse in Senegal; seemingly caused by bit mouth-piece.

**Figure 2:**
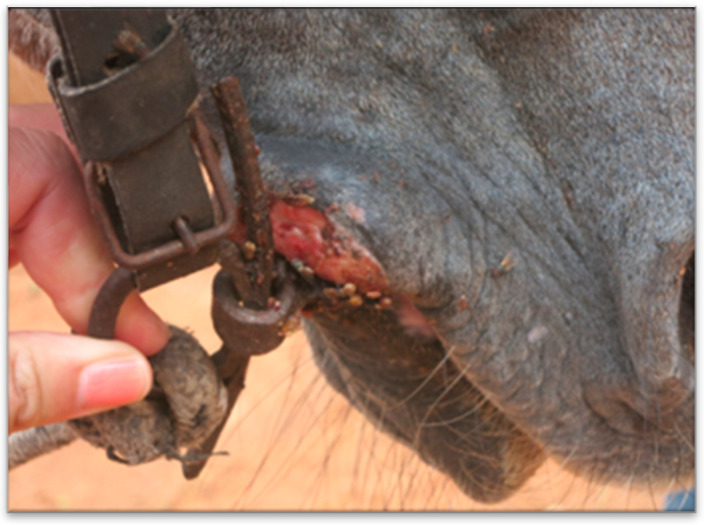
Bit-related lip lesion on a cart horse in Senegal; seemingly caused by bit cheek-piece.

**Figure 3:**
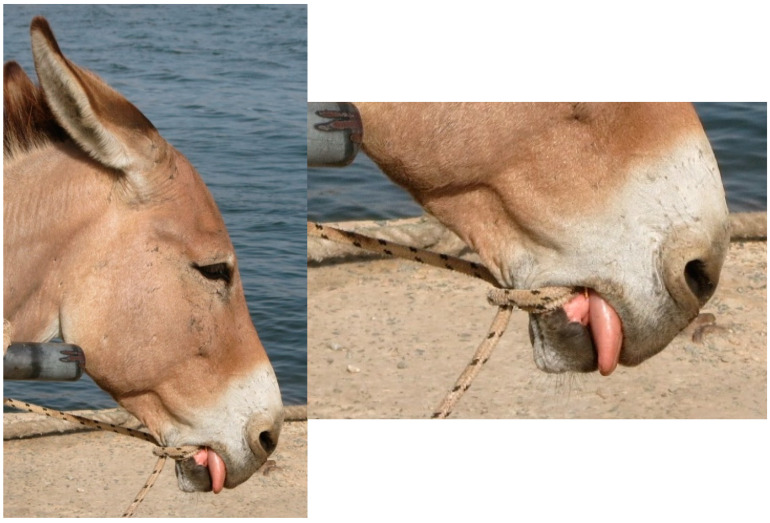
Use of rope around lower jaw in place of a bit in a cart donkey in Senegal.

**Figure 4:**
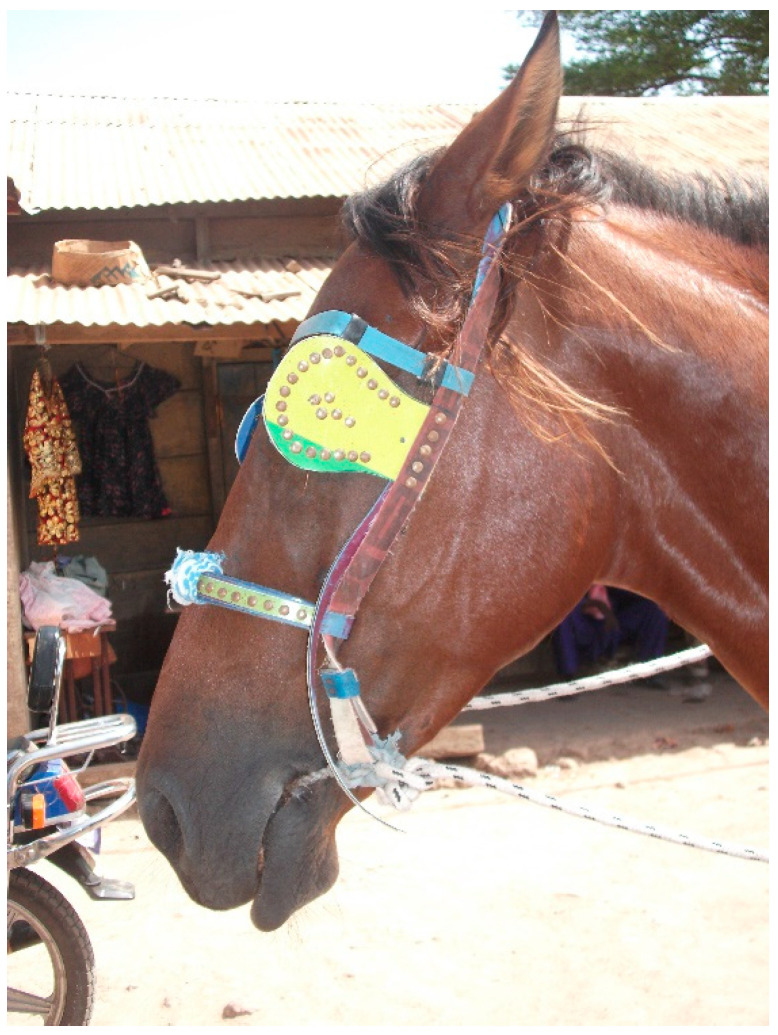
Use of rope in place of a bit in a cart horse in Senegal.

Welfare data collected within Brooke West Africa’s operational areas using Brooke’s Standardised Equine-Based Welfare Assessment Tool (SEBWAT), coupled with direct observation of working equids in Senegal by field staff, indicated the widespread presence of lip lesions (Figure 1 and Figure 2) and oral injury amongst working equids involved in transportation of people and goods by cart. SEBWAT comprises 40 animal-based indicators for working equid welfare assessment [6], including a specific measure for assessing lip lesions ([7] adapted from [8]). (For more information about this and other animal-based welfare indicators and assessment protocol used internationally by Brooke, see www.animalwelfareindicators.thebrooke.org (accessed on 10 August 2022).) According to SEBWAT data collected by Brooke West Africa in 2014–2016 (data available on request from corresponding author), based on assessment of 965 working equids across Brooke five project areas, prevalence of lip lesions ranged from 19% to 27%. Several probable causes of oral injuries were identified, including the practice of passing rope through the mouths of horses and donkeys (Figure 3 and Figure 4), causing injury at the sites of rope contact with the skin; and driving methods that appear likely to create oral trauma, such as application of strong force through the reins and bits—sometimes sufficient to displace the bit laterally through the mouth—or sudden, abrupt jerking motions on the reins during work (M. Seck and A.F. Brown, personal observations). Bit-related oral injury has been extensively reported in the literature, including in ridden horses in Sweden [9], Danish and Islandic competition horses [10,11,12], horses and ponies competing in cross-country events [13], Finnish trotters [14,15] polo ponies and race-horses [16]. These published findings were commensurate with field observations by Brooke West Africa welfare assessors and programmatic staff indicating that lip lesions in working horses in Senegal appeared to be predominantly bit-related. Furthermore, field staff commonly encountered drivers who said they provided what they perceived as adequate feed to their equids, yet observation of the equids revealed poor body condition and animals apparently struggling to eat properly due to oral pain seemingly making mastication uncomfortable or difficult.

Contemporary bits typically constitute a cylindrical bar of metal that is placed within the mouth of the equid during work (although historically, when early bits were introduced, they are likely to have been made of leather, bone or wood [17]). The bit is held in position by the bridle’s cheek-pieces on either side—narrow straps of material that extend from one end of the bit to the other, passing over the poll (the highest point of the equid’s head, behind the ears) [18,19]. Reins are attached to each end of the bit and held by the hands of the rider, driver or handler. The reins and bit in conjunction provide a means of communication to control the speed and direction of the equid’s movement by transferring pressure from the person’s hands, along the reins and onto the animal’s head via the bit and bridle. Many varieties of bits are commercially produced, and are made of steel, copper, rubber, plastic or occasionally leather (see [20,21,22] for examples of commercially available bit designs). There are many differing mouth-pieces (e.g., straight, curved, twisted, jointed, with multiple links, lozenge, port, rollers) and bit cheek-pieces (e.g., D-ring, eggbutt, full cheek, half cheek, pelham, gag, shank, Kimblewick).

The use of bits is now commonplace wherever domesticated equids exist around the world, and archaeological evidence indicates the use of bits on horses as long ago as 1200–1300 BC [23,24]. However, whilst bits are a prevalent and broadly accepted component of equestrian tack, they can impair equid welfare by causing discomfort, pain, injury and behavioural inhibition, and can also inhibit breathing and locomotion [25,26,27,28,29,30]. Bits, as a component of the overall bridle, function by exerting pressure on the bars of the mouth, tongue, lip commissures, hard palate, poll, chin and nose, with some variation in action according to the bit and bridle type [31,32,33]. As sensitive structures, these parts of the equid’s head and oral cavity are vulnerable to pain and injury if subjected to excessive, prolonged or repeated pressure via the reins and bit, and research indicates behaviours indicative of aversion (including bite marks on bits where equids have grasped them between the teeth) and oral pain associated with bitting [33,34,35]. The manner of usage of bits and bridles is dependent upon the knowledge, skill and attitude of the person holding the reins, and of those who produce, select and fit the bit and bridle. Accordingly, when knowledge of lorinery and skills in riding/driving are lacking, or rider/driver attitude is not sensitive to the equid’s physical and behavioural welfare needs, there is scope for harm to be caused by inappropriate use of bits. Welfare impairment can also arise when the design of the bit or bridle is inherently unsuitable or harmful, e.g., a very high port that places pressure upon, and causes injury to, the hard palate during use [36]; is mismatched to the individual equid, e.g., bit has not been changed as the equid ages to accommodate changes in teeth angulation and oral dimensions over time [31,37]; or is used poorly by people, e.g., tight nose-bands which exacerbate bit effects and associated injuries [11].

Lorinery (originally, the craft of producing bits and bridles, as well as other metal components of tack such as stirrup irons and buckles [38]) is a specialist subject, and learning opportunities and resources on lorinery are not readily accessible to working equid owners, users or animal health and welfare practitioners in Senegal. Many owners and users of working equids in Senegal have learned about harnessing only from their peers or previous generations [39]. Whilst traditional knowledge and ‘word-of-mouth’ can be valuable means of conveying important information, the lack of formal training and standardisation causes many defective bits to be used. Furthermore, even in locations and communities of practice with access to learning materials and training on equestrian harness, including lorinery, equid welfare is still compromised by harmful use of tack or aids [30], such as over-tightening of nose-bands in eventing and dressage [40]; use of ‘crank’ nose-bands, which have been associated with increased physiological stress responses and impaired blood circulation [41]; or the use of tongue-ties in Thoroughbred and Standardbred racing, which can alter the equid’s airway [42,43,44].

**Figure 5:**
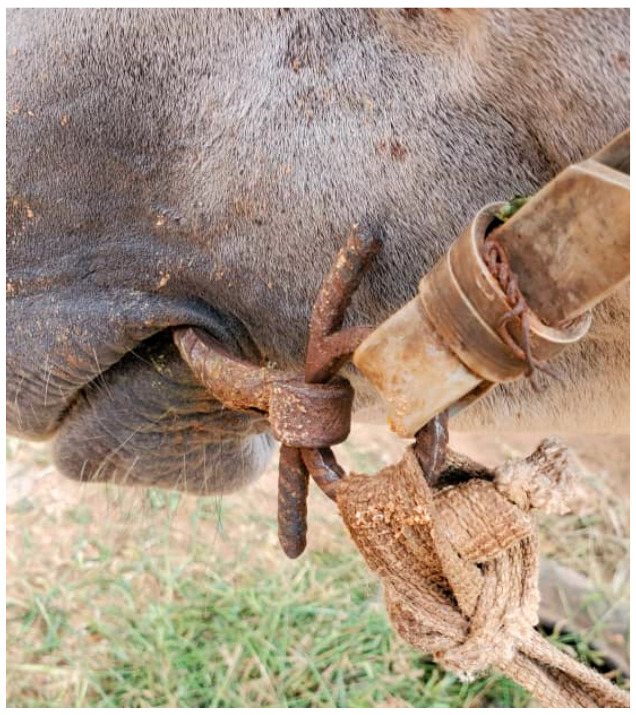
Locally produced bit in Senegal with ridged surfaces and protruding cheek-pieces that contact the skin of the horse’s face.

**Figure 6:**
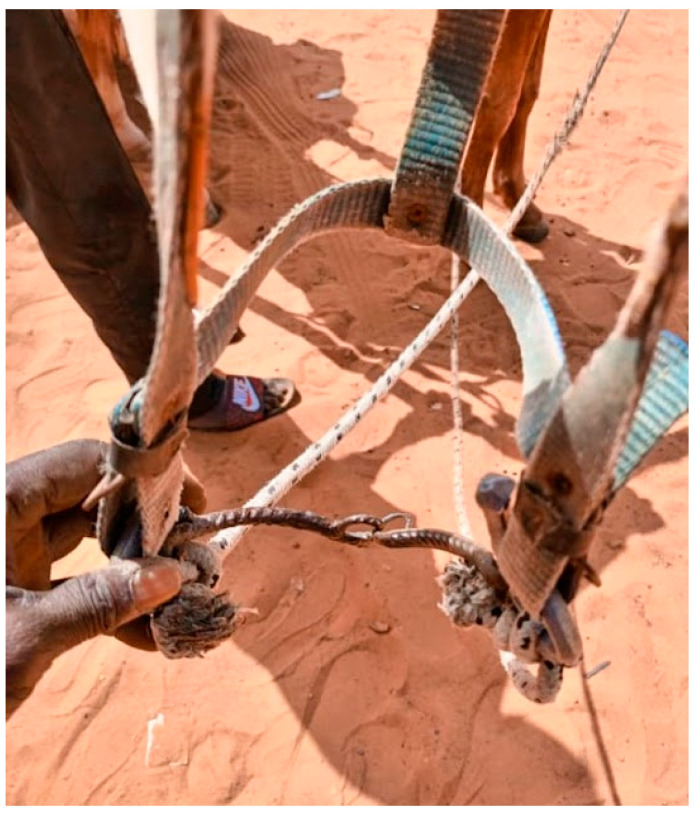
Locally produced bit in Senegal made from narrow pieces of construction iron with ridged surfaces.

**Figure 7:**
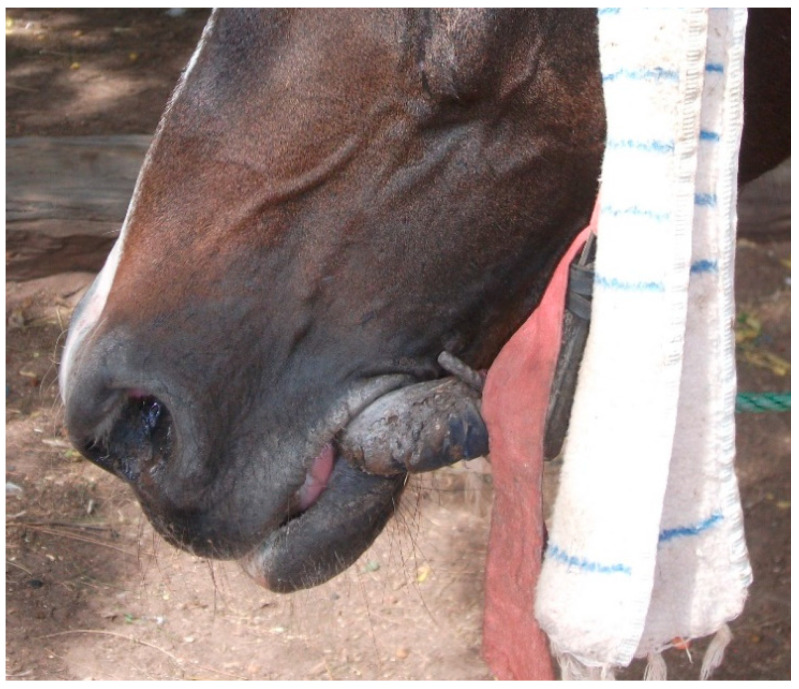
Locally produced bit in Senegal with excessively thick mouth-piece inhibiting mouth closure.

**Figure 8:**
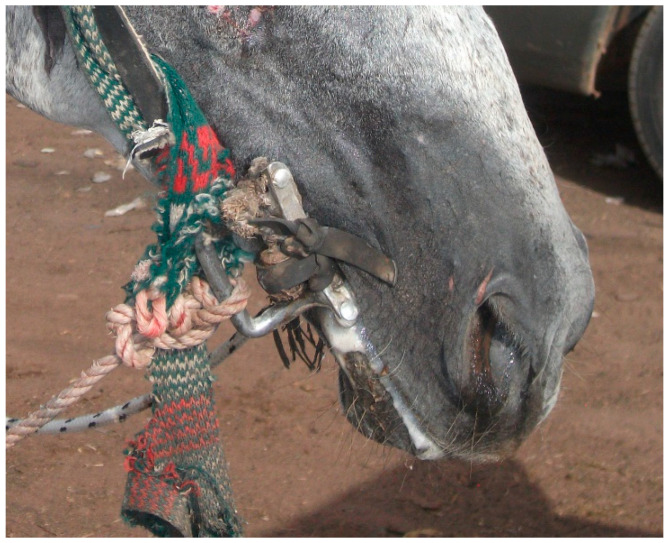
Locally produced bit in Senegal with cheek-pieces pressing against the equid’s face, and multiple knots and attachments which are a potential cause of discomfort, pressure and altered fit.

The majority of the bits currently used for working equids in Senegal are made of construction iron, recovered from the rubble of destroyed houses or near construction sites (M. Seck, personal observation). The bits are typically designed and produced by local blacksmiths who inherited the knowledge from their parents, and often have defects related to their design, shape and quality of the metal from which they are made. For example, the construction iron has asperities and rusts quickly, and the metal may be twisted or have a ridged surface (Figure 5 and Figure 6), all of which may increase friction during abrasion against the equid’s soft tissues. The mouth-piece is typically straight and of variable thickness (diameter)—in some cases very narrow (Figure 6), which is known to increase risk of injury [15], and in other cases so thick as to prevent closure of the mouth (Figure 7). There is also variation in bit cheek-pieces, which might have protrusions that press against the skin of the equid’s face (Figure 5 and Figure 8), in some cases exacerbated by the mouth-piece being too short for the equid’s mouth or an assortment of knots and attachments creating an additional source of discomfort (Figure 8).

The locally produced bits are generally sold in marketplaces at a relatively low cost of 500 CFA (equating to approximately $0.8–1 USD). Bits imported from Europe are also available within Senegal, but these are mostly reserved for equestrian sports and race-horses as the cost is prohibitively high for working equid communities at 10,000 CFA (equating to approximately $15–20 USD).

As equids involved in traction and transport often work for many hours each day or week, the welfare impacts of their tack in general, and bits in particular, have potential to affect them on a near-daily basis and for multiple consecutive hours. Thus, the longevity of the welfare impairment associated with tack and bits is an important consideration as well as its severity. Cart horses in Senegal typically work from 06:00–13:00 and 16:00–22:00 in the dry season when the temperatures are relatively low (November–February); and 06:00–13:00 and 16:00–20:00 at the end of the dry season and during the rainy season (March–October). Research from Senegal has indicated carriage horses transporting people make as many as 30 daily trips, and cart horses transporting goods carry loads of 400–800 kg [5]; and it has been suggested that ‘heavier’ work increases welfare risks to working equids [45]. Equids involved in draught work have also been found to have greater prevalence of lip lesions than those involved in pack work types [46], further supporting the prioritisation of cart horses for the present study.

In addition to acute and recurrent welfare impairment whilst wearing the bridle, there is also risk of welfare impairment that affects the equid beyond the time spent wearing the bridle, and of cumulative welfare impacts over time. For example, the equid may learn to associate wearing the bridle with pain and discomfort, and experience negative anticipation, fear or distress prior to or during daily tacking up. Ridden horses have been found to exhibit behaviours associated with stress or pain during tacking up, which has been interpreted as indicating their anticipation of stress or pain during subsequent activities [35]. This is an example of the equid learning, through classical conditioning, to associate the oral pain experienced or exacerbated when wearing the bridle (i.e., the unconditioned stimulus), with the approach of the handler and bridle (i.e., the conditioned stimulus). Therefore, fear of the pain may be triggered in anticipation of the negative experience to follow and reinforced as an appropriate psychological response, from the animal’s perspective, when the application of the bridle does repeatedly cause pain [47].

As pain, fear and stress are considered aversive mental states [48,49,50] welfare is impaired as a consequence. Whilst questions have been raised about the functional basis of positive anticipatory behaviour and the reliability of measuring this and drawing welfare inferences [51], there is evidence that anticipatory behaviour occurs in various vertebrate species and differs in response to a positive or stressful conditioned stimulus [52,53,54], suggesting a possible impact on welfare of anticipating an aversive event.

Oral injury and tissue damage have potential to impair the equid’s ability to graze, chew and obtain sufficient dietary intake, for example due to quidding or failure to consume enough feed in the limited time available for eating around work requirements. Such occurrences may predispose to loss of body condition and associated secondary implications, including increased susceptibility to skin lesions [8] and the associated risk of localised or systemic infection with pathogens entering via broken skin. Masticatory inefficiency is associated with gastro-intestinal ailments including choke and colic (e.g., as a consequence of dental disorder [55,56]) which can be life-threatening for equids. As malnutrition is associated with immunocompromise [57], equids who lose condition due to inadequate nutrition will also be more vulnerable to disease. Working equids may already be at high risk of disease due to lack of preventative measures, and limited access to animal health practitioners, who are often scarce or unaffordable in many working equid contexts [58].

In light of the prevalence of bit-related injury, potentially significant welfare impairment for affected working equids and lack of local alternatives to current bitting practice, Brooke West Africa devised a pilot project to manufacture and trial improved bits for horses working in transportation of people and goods, in five project areas. The aim of the study was to develop a locally made prototype of an improved bit design for this group of working equids, trialling its efficacy as a less harmful bit for the horses and its acceptability as an alternative piece of equipment to equid owners/users in the target demographic. The overall goal, in line with Brooke’s mission of working equid welfare improvement, was ultimately to evaluate the welfare benefits and feasibility of implementing interventions that encourage uptake of less harmful bits amongst working equid owners/users more widely throughout Senegal, and beyond in the wider West Africa region wherever similar bitting problems exist.

## 2. Methods

### 2.1. Design of Improved Bits

Based upon the materials available locally, two types of bits were initially designed for testing: plastic-coated iron bits and aluminium bits. There was a tendering process involving five artisans who were initially selected from a cohort of Brooke-trained farriers who had demonstrated a high level of skills in forging and metalwork, and were therefore invited to submit bit prototypes for tender. This approach prevailed because there were no other people known to the Brooke West Africa team who were considered sufficiently skillful for the anticipated work, and these individuals already had an established effective working connection with Brooke and were cognisant of the charity’s mission and aims regarding working equid welfare improvement. All artisans participated in the tender process on a voluntary basis, worked with their own funds and were not paid during this phase of the process. Firstly, a written call for applications, including images of the bit specifications, was distributed, then a meeting was organised with the applicants to ensure that all had a common understanding of the work. During this meeting, technical specifications on the material, bit mouth-piece, bit cheek-pieces, thickness and length of the mouth-piece (Table 1) were discussed with the artisans. These technical specifications were determined based on review of available literature and information sources on lorinery, including academic and equitation texts and guidance provided by bit manufacturers and retailers (accessed via retail websites), coupled with existing knowledge of bitting from practical experience in equitation (A.F.B). The aim was to minimise bit severity and risk of harm to the equids should drivers use them in a ‘heavy-handed’ manner (such as applying strong, sudden or severe pressure on one or both reins), whilst accepting the limitations of what is feasible to produce locally with the materials, tools and skills available.

Each artisan then submitted two initial prototypes: a plastic-coated iron bit and an aluminum bit. Feedback was provided (from A.F.B. based on review of photographs and videos) on improvements to be made to these initial designs with the aim of mitigating discomfort and reducing severity of action for the horses. Examples included requesting the removal of long shanks on cheek-pieces which risked creating excessive leverage and poll pressure during use [32], high ports on mouth-pieces which risked contacting the hard palate [59], and sharp or abrasive surfaces which appeared likely to increase friction against oral tissues. Artisans were allocated an additional four weeks to refine and finalise the design of the prototypes. Prior to submission, artisans independently undertook some informal trialling of the prototypes with cart horses to assess the absence of harm and functionality, and photographs and short video clips of the bit prototypes were sent to Brooke staff for remote evaluation. (As the tendering artisans were also farriers who had participated in Brooke West Africa trainings for several years, including training in animal welfare, equine behaviour and handling, they were deemed capable of identifying presence or absence of harm to equids associated with the bit trials, such as behavioural resistance or oral injury.) All artisans also sent their two bit prototypes to the Brooke office in Dakar for the final evaluation based on the technical specifications provided. The compliance on some criteria (comfort in mouth, clarity in signal, ability to turn) was confirmed by testing the bits with 11 horses near the Brooke office in Dakar. The artisan’s financial offer (i.e., cost per bit) was also considered to ensure the product price would be feasible in the local context. Finally, one artisan successfully met all of the requirements and was contracted to reproduce his bit prototype on a larger scale. This artisan received payment for producing his bits for both the testing phase (30 bits) and subsequent experimental phase (1000 bits).

### 2.2. Development of Animal-Based Indicators

Several animal-based welfare indicators were designed with the aim of measuring potential positive or negative effects of the new bits, comprising six behavioural indicators and four physical indicators, the latter for pertinent oral lesions (Table 2). Of the behavioural indicators, licking/chewing motions in the absence of food and tension in the facial muscles were observed whilst the horse was static, as it was considered that these may reflect the horse’s response to a foreign body in the mouth, or indicate discomfort or stress, respectively [34,63,64,65]. Opening of the mouth, extension of the tongue out of the mouth, and shaking, tossing, tilting or turning head movements were observed during locomotion, as these behaviours are considered indicative of evasion of, or resistance to, bit pressure [27,33,47,63,66,67]. The physical indicators reflected sites of potential injurious contact between bits and oral tissues, where pressure or pinching may occur whilst the bridle is in use [16,19,46,68,69].

### 2.3. Testing Phase

30 drivers in Thiès (a municipality in the west of Senegal) who used working equids for the transportation of goods or people by cart, were selected to test the new bit prototypes (14 plastic-coated iron bits with half cheek-pieces and 16 aluminium bits with D-ring cheek-pieces, both with mullen mouth-piece) with their horses. This location was selected due to proximity to Dakar, and the known presence of horses involved in cart transportation work. The sample size of 30 for the testing phase was chosen according to time allocated to the project, and the assessment that it would be sufficient to check for the absence of harm before scaling-up, whilst not involving more horses than necessary to gain this reassurance. Drivers were recruited via prior discussion and outreach from the local community-based partner, or via direct discussion in situ between Brooke staff, partners and drivers. All drivers were male (as women do not commonly drive horses in Senegal), participated in the testing phase on a voluntary basis without payment, and were fully informed of the study aims, process and requests of them/their horses prior to verbally consenting to participate along with their horses.

The demographic information of owners and horses is summarised in Table 3. Criteria for inclusion of drivers/horses in the testing phase were as follows.

1. Voluntary participation of drivers. This was necessary as drivers had to be involved in the monitoring process. After a short training, they were advised to be vigilant for any signs of pain/discomfort in their horses, to remove the bits in that case and inform the Brooke team without delay so that other drivers could be alerted to discontinue use of the same bit design. This approach was chosen to mitigate animal welfare risks. The training, which was mandatory for all participants in the study, consisted of recognition of signs of pain and discomfort caused by the bits so that they could report to Brooke West Africa team. This occurred in a classroom environment (at a partner organisation’s headquarters). Drivers were asked to explain using their own words how they recognise signs of pain and discomfort in their horses. The majority described this well in accordance with expectations, and with the few who demonstrated some differences in understanding signs of discomfort, PowerPoint and video presentations helped them to achieve the correct understanding.
2. Work of horses on a daily basis. Horses engaged in commercial transportation of people or goods were selected due to the frequency of work, and thus ability to appraise effects of the bits over a relatively short time period (as compared, for example, to trialling with horses who only work once per week).

Criteria for exclusion of drivers/horses from the testing phase were as follows.

1. Not working in transportation of people or goods by cart. Other work types (e.g., agriculture, pack transportation) were excluded as the horses were not used frequently, which could lead to misleading results (e.g., absence of lesions due to the rarity of bit use rather than the quality or design of bits).
2. Drivers younger than 15 years of age. Juvenile drivers were excluded for ethical and child protection reasons and to comply with Senegalese national legislation, which prohibits the driving of horses by people of this young age group.
3. Horses in poor physical condition. This included horses with a body condition score less than two, observable symptoms of illness or disease, lameness or body lesions with broken skin. Such animals were excluded for ethical reasons, to avoid any unnecessary intervention that may disturb them, in compliance with Brooke’s international animal welfare policy and ethical standards for research.

The testing phase occurred in one municipality and with a relatively small sample of 30 drivers/horses because the purpose of this phase was predominantly to appraise potential animal welfare risk associated with the alternative bit designs, and ensure any emergent risks could be thoroughly mitigated prior to and throughout the subsequent experimental phase. Involving a larger number of subjects risked avoidable harm should the new bits lead to welfare impairment, or danger for drivers and/or horses if the new bits led to difficulty with safe control of the horses’ speed or direction during work. It was important to avoid the risk of introducing a new bit type that was subsequently found to be suboptimal and not being able to retrieve distributed bits from a larger trial group. Similarly, the risk of other drivers (not participating in the testing phase) assuming the new bits are good alternatives prior to adequate testing, and potentially replicating bit designs that were subsequently identified as unsuitable or suboptimal, was also important to avoid.

Data collection occurred at the horses’ usual places of work, during resting times in their normal working schedule, between the hours of 13:00–16:00. Commensurate with Brooke’s international animal welfare policy, efforts were made to optimise horses’ comfort through seeking shade when possible. A discussion on the objectives and methodology was held with the driver after which he decided whether or not to participate in the activity on a voluntary basis.

The two assessors involved in animal welfare data collection had received previous training and certification in Brooke’s Standardised Equine-Based Welfare Assessment Tool, including in equid handling and behaviour (see www.animalwelfareindicators.thebrooke.org/welfare-measurement/how-do-we-train-assessors (accessed on 10 August 2022) for more information about this training), and were experienced in conducting working equid welfare assessments in similar field conditions. Both were Senegalese veterinarians, had five and three years of experience respectively of working equid welfare projects, and were familiar with the culture and language in the study location. Prior to commencing data collection, they received an additional two-hour preparatory training (from M.S.) focussed on correct administration of the drivers’ questionnaires, and a standardisation exercise to maximise scoring consistency. The baseline and endline data from horses and drivers were collected by the same two assessors on both occasions.

Scoring criteria for the behavioural and physical parameters are described in Table 2. The horses were evaluated during their usual resting time (of two-three hours), during which bridles were removed as normal. The driver was asked to replace the bridle with the pre-existing bits and each horse was first observed with this fitted as normal, and two behavioural parameters (lick/chew and facial tension) noted whilst the horse was static for a period of two-three minutes. The four physical parameters (lesions on lip commissures, tongue, buccal mucosa and bars) were also recorded during this static phase. In order to assess these, the assessors calmly approached the horse with the assistance of the driver for handling if necessary. The bridle was removed then the assessor opened the mouth gently using the thumbs (as per protocol described in [70]) in order to observe the relevant oral tissues. The mouth was observed for the presence or absence of signs of injury on each of the four areas.

The pre-existing bit was then removed from the bridle, replaced on the same bridle by the new bit, and the bridle re-fitted onto the horse. Drivers fitted the replacement bits themselves, as they were familiar with their individual bridles, under the supervision of the assessors who were also present to observe for signs of discomfort or pain from the horses, and oversee handling and welfare standards throughout. A two-three-minute period of adjustment was included to allow the horse to familiarise with the feel and taste of the new bit. After the period of adjustment to the new bit, the same two behavioural parameters (lick/chew, facial tension) were recorded whilst the horse was static as described above. The horse was then driven forwards at a walking pace for three minutes, and the four other behavioural parameters recorded during this locomotion phase (mouth open, tongue loll, head toss/shake, head tilt/turn).

This initial data collection occurred in October 2020, and after three weeks, in November 2020, the same behavioural and physical parameters were recorded again. This was during the end of the wet season in Senegal, and temperatures were between 30–38 degrees centigrade. A time period of three weeks was selected for the testing phase as it was considered that this would be sufficient time for oral lesions due to harmful bits to occur, or to be reassured that they would not occur, when horses were worked in their bridle on a daily basis, based on previous experience of Brooke veterinarians and field staff in Senegal.

Drivers were requested to be continually vigilant to their horses’ behaviour when driving, monitor on a daily basis for absence of pain and/or discomfort, and to verify the presence of any lesion, scab or bruising each day after removing the bit/bridle throughout the three-week trial period to determine efficacy of the new bits and ensure welfare risks were mitigated prior to considering any scaling up for the experimental phase. Participants agreed to contact the assessors by phone to describe their findings and seek advice if necessary, and assessors were also able to request videos from the participants if further clarification was required. Assessors were able to maintain a suitable level of individual engagement with drivers and monitoring of the testing via communication with mobile phone, which would have been more challenging with a larger trial group.

Feedback from the drivers who had conducted daily monitoring suggested that the new bits had not created any negative behavioural or physical impacts for the horses, nor any controllability difficulties for the drivers. Furthermore, the behavioural and physical indicators had shown improvement between the beginning and end of the testing phase, after the pre-existing bits had been replaced with the new designs (Table 4).

**Figure 9:**
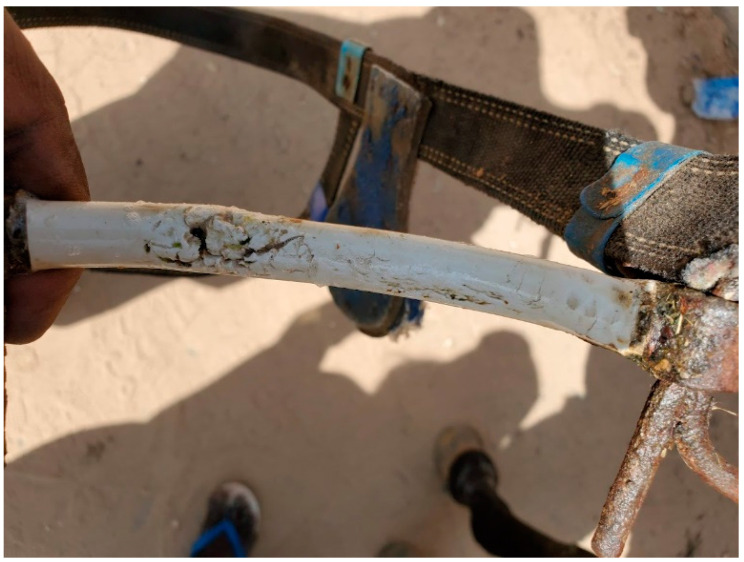
Damage occurring to plastic coating during testing phase; hence removal of this prototype from further trialling and scale-up.

Although there was no evidence of physical damage occurring to the horses, following the three-week trial the decision was made to abandon the plastic-coated iron bit prototype because this did not offer resistance guarantees, with six out of 14 of this prototype incurring cracks (Figure 9). It was thus considered that the cracked plastic coating presented a welfare risk to the horses, as the oral tissues may be susceptible to pinching, abrasion or cutting by the cracked plastic. Therefore, the plastic-coated iron bits were removed from the study after the testing phase, and only the aluminum bits with D-ring cheek-pieces were used during the subsequent experimental phase.

The experimental phase started three months after completion of the testing phase due to delays caused by the COVID-19 pandemic and the time needed to establish a contract with the successful artisan to produce 1000 bits. 540 of these were to be used in the experimental phase, and the remainder retained for subsequent selling as it was intended to gauge feasibility of sale as an income-generation activity for local vendors.

### 2.4. Experimental Phase

**Figure 10:**
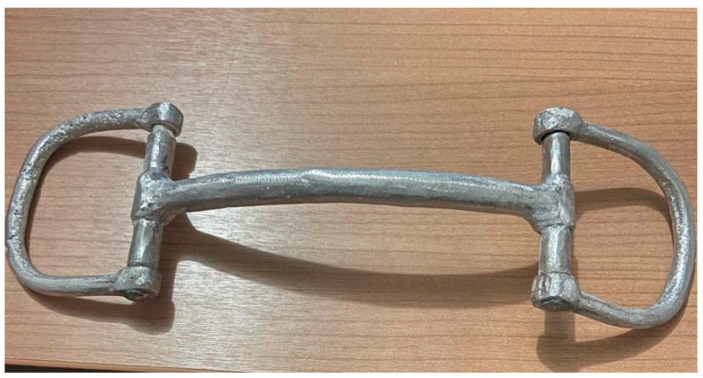
The successful bit prototype selected for trialling during the experimental phase.

**Figure 11:**
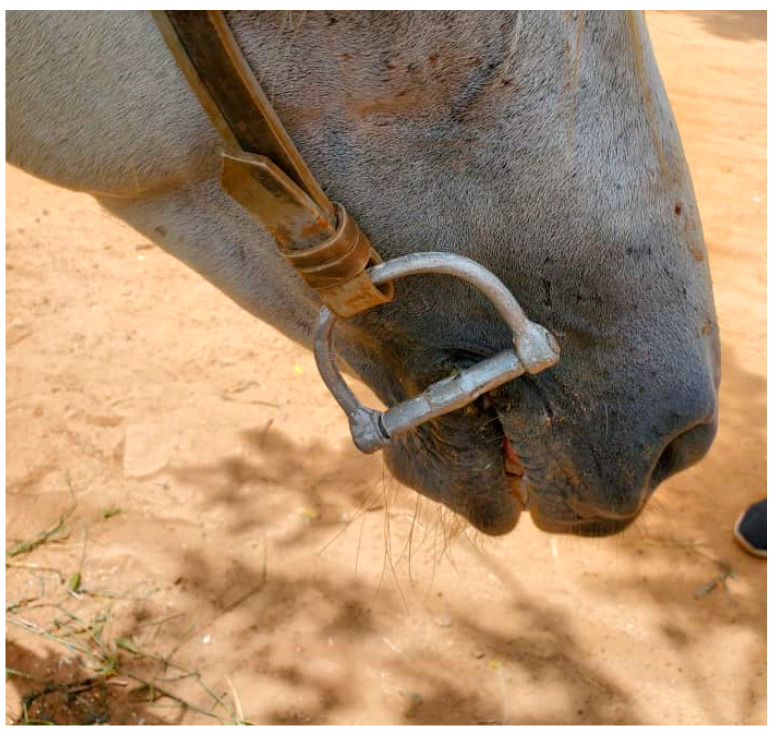
The successful bit prototype fitted to a cart horse’s bridle.

After the testing phase, which provided reassurance that the selected bit design had not been found to instigate any harm, animal welfare risk or driving difficulties—and indeed had even enabled pre-existing oral injuries to heal despite continuation of usual daily work throughout the trial period—the production of the selected bit design (i.e., aluminium bit with mullen mouth-piece and D-ring cheek-pieces; Figure 10 and Figure 11) was increased to enable the experimental phase to be conducted with a greater number of drivers and horses. Five municipalities in Senegal where Brooke operates were selected for the experimental phase: Thiès, Mékhé, Louga, Bambey and Sokone. These locations were selected to enable implementation of the study and successful follow-up as Brooke had established partners in these areas who had existing knowledge of the cart horse drivers and could facilitate engagement. Additionally, as scale-up of the new bits was anticipated, these local partners had an important future role in promoting and encouraging uptake of these new bits should they prove successful, therefore their active participation in the experimental phase was an important precursor to intended subsequent stages of Brooke’s project work.

A total of 540 drivers/horses (100–120 in each area) were selected; each driver worked with one horse, so the numbers were equal. The same criteria for inclusion and exclusion of drivers/horses as previously described for the testing phase were applied in the experimental phase, with the addition of: (a) excluding drivers who were subject to seasonal migration, to ensure continuity of the study and minimise attrition losses; and (b) excluding drivers with less than one year driving experience, to minimise the risk of poor driving skills leading to oral trauma independently of the quality of the bits. As for the testing phase, drivers were recruited with the support of local, community-based partner organisations, and informed consent obtained verbally (due to low literacy of participants) prior to their involvement in the study. The new bit prototypes were distributed to the participant drivers without charge in return for their participation in the study, and no payment was made to or from either party. The experimental phase involved a convenience sample of drivers who met the inclusion/exclusion criteria and were willing to participate, as their voluntary participation and willingness to monitor and provide feedback to the assessors was necessary for successful implementation of the study. It was also important to ensure continued contact with the cohort of drivers throughout the study period to enable adequate follow up and liaison in the event that any negative welfare effects were emergent at any time, or drivers required advice from Brooke staff for any reason. Participants were willing to engage with the Brooke team and it was possible to maintain telephone contact throughout the study period.

The new bits were distributed in March 2021, and data collected for behavioural and physical parameters as described for the testing phase, aside from exclusion of the behavioural data related to the old bits. Data pertaining to the old bits were not collected because the purpose of the experimental phase was to assess the effect of the improved bits over a longer period, with short-term efficacy and mitigation of negative consequences already having been satisfactorily established during the testing phase. A repeat data collection of the same parameters was conducted 21 weeks later, in July 2021. The total duration of the process was 21 weeks in order to provide a medium-term view of the impact of the improved bits on the welfare of the working horses. Data were collected by the same two assessors involved in the testing phase as previously described, with logistical support from the community-based partners who were also responsible for retracing the drivers involved in the study.

### 2.5. Data Analysis

The demographic data were reported as percentage frequencies calculated using Microsoft Excel. The experimental data were analysed using SPSS software for Windows, version 1.0.0.1406. McNemar’s test (set at 95% confidence) was applied to each of the behavioural and physical parameters to explore differences between data taken in March, at the beginning of the experimental phase, and data taken in July, at the end of the experimental phase.

### 2.6. Ethical Approval

Prior to commencing any data collection, ethical implications for animal and humans were considered and mitigated by the authors in accordance with Brooke’s AWERB Guidelines for Researchers (available at https://www.thebrooke.org/sites/default/files/Downloads/Final%20AWERB_Guidelines_v6.pdf (accessed on 10 August 2022)) and Brooke’s Good Research Practice guidance (available on request from the corresponding author). As with all Brooke-funded work, the study was also required to adhere to relevant sections of Brooke’s international animal welfare policy which aims to “*minimise animal welfare risk and promote positive animal welfare throughout all Brooke activities*”. The study was exempt from formal licensing processes in Senegal.

## 3. Results

### 3.1. Descriptive Data

Data describing the demographics of the drivers and horses included in the experimental phase are detailed in Table 5. All of the participants were male, reflecting the cultural norm in the study location for cart horses to be almost exclusively worked by men, and the majority had more than five years of driving experience. They are typical of the broader population, with the exception of the driving experience, as drivers with less than one year driving experience were deliberately excluded. The majority of the horses transported people by cart and were between three and 12 years old. All of the horses were stallions as is consistent with local practice to only drive stallions.

### 3.2. Physical Parameters

#### 3.2.1. Lip Commissures

When assessed in March at the time of introducing the new bit prototype, 211 of the 540 horses were observed to have injury at the lip commissures, compared to 23 horses in July; an 89% decrease in the proportion of observed injury at the lip commissures. McNemar’s test indicated that the change between March and July datasets was significant (*p* < 0.001).

#### 3.2.2. Tongue

When assessed in March at the time of introducing the new bit prototype, 103 of the 540 horses were observed to have injury to the tongue, compared to two horses in July; a 98% decrease in the proportion of observed injury at the tongue. McNemar’s test indicated that the change between March and July datasets was significant (*p* < 0.001).

#### 3.2.3. Buccal Mucosa

When assessed in March at the time of introducing the new bit prototype, 88 of the 540 horses were observed to have injury to the buccal mucosa, compared to one horse in July; a 99% decrease in the proportion of observed injury at the buccal mucosa. McNemar’s test indicated that the change between March and July datasets was significant (*p* < 0.001).

#### 3.2.4. Bars

When assessed in March at the time of introducing the new bit prototype, 105 of the 377 horses were observed to have injury to the bars of the mouth, compared to zero horses in July; a 100% decrease in the proportion of observed injury at the bars. McNemar’s test indicated that the change between March and July datasets was significant (*p* < 0.001). The smaller dataset for bars as compared to the other parameters occurred because there were occasions when the horses were resistant to opening their mouths, so the assessors were unable to make reliable observations. (This applied particularly in Sokone and Mékhé locations where assessors reported that horses involved in transportation of people by cart showed more avoidance behaviour.) As animal welfare remained paramount in accordance with Brooke’s policies, assessors did not persist with multiple attempts to open the mouth when strong resistance or signs of distress were shown by the horse.

### 3.3. Behavioural Parameters

#### 3.3.1. Open Mouth

When assessed in March at the time of introducing the new bit prototype, 58 of the 540 horses were observed to have their mouth open, compared to three horses in July; a 95% decrease in the proportion of horses displaying this behaviour. McNemar’s test indicated that the change between March and July datasets was significant (*p* < 0.001).

#### 3.3.2. Tongue Loll

When assessed in March at the time of introducing the new bit prototype, 27 of the 540 horses were observed to have their tongue visible, compared to five horses in July; an 82% decrease in the proportion of horses displaying this behaviour. McNemar’s test indicated that the change between March and July datasets was significant (*p* < 0.001).

#### 3.3.3. Head Toss/Shake

When assessed in March at the time of introducing the new bit prototype, 45 of the 540 horses were observed to demonstrate head toss/shake, compared to three horses in July; a 93% decrease in the proportion of horses displaying this behaviour. McNemar’s test indicated that the change between March and July datasets was significant (*p* < 0.001).

#### 3.3.4. Head Tilt/Turn

When assessed in March at the time of introducing the new bit prototype, 34 of the 540 horses were observed to demonstrate head tilt/turn, compared to five horses in July; an 85% decrease in the proportion of horses displaying this behaviour. McNemar’s test indicated that the change between March and July datasets was significant (*p* < 0.001).

It should also be noted that none of the drivers in the study had reverted back to using the previous bit type, nor did any report difficulties with safe control of their horses when using the new bit types.

## 4. Discussion

It was hypothesised that making improvements to bit design would lead to amelioration of bit-related welfare issues amongst working cart horses. The results exceeded expectations, indicating statistically significant improvements in all of the animal-based indicators measured.

As with many facets of equestrianism, such as farriery or saddlery, much current knowledge and practice on lorinery has its roots in traditional techniques, and is often predicated upon replicating the actions of peers, forefathers and word-of-mouth, as opposed to empirical science. Whilst peer-reviewed science and evidence-based practice is growing in equine science and equestrian topics, such as farriery (e.g., [71,72,73]) and equine training methodology (e.g., [74,75,76,77]), a contemporary evidence base of peer-reviewed science for lorinery, and harnessing more broadly, remains relatively scarce. It is encouraging to see greater focus in recent years on the welfare implications of equid harnessing, aids and related aspects, such as the impact of nose-bands on the welfare of performance horses [78,79] or of whips in racing [80,81,82]. However, there remains a paucity of literature and research on these topics in relation to working equids specifically, perhaps due to their presence being predominantly limited to resource-poor contexts or low- and middle-income countries where there are other research priorities; or the lack of institutional donors prepared to fund such research when it may not have direct implications or benefits for the demographic or geographical location where their focus lies. Accordingly, much of the existing research on working equid welfare has been conducted and funded by international animal welfare charities, such as Brooke, the Donkey Sanctuary, World Horse Welfare and Spana. Although there are valuable contributions by researchers based in countries where working equids make fundamental contributions to livelihoods, communities and national economies such as Ethiopia, Pakistan or Egypt [83,84,85,86,87,88,89,90,91], working equid welfare continues to be under-represented in the literature (hence the present authors’ desire to publish these findings in order to contribute to filling this gap and supporting other working equid welfare practitioners).

Whilst the physical and behavioural aspects of welfare that are influenced by bits are only a portion of those that make up the equid’s daily and lifetime experience, they are significant in that they typically impact upon working equids on a near-daily basis and throughout their working life, which may extend to as many as 20 years. Despite the obvious importance, bitting of working equids has not been well-researched. As working equids typically endure a plethora of welfare problems including body lesions, hoof problems, dehydration and malnutrition, ectoparasites, lameness and chronic pain [8,47,92,93,94,95,96,97], it is possible that bit-related harms may be overlooked by practitioners and researchers, either due to being perceived as of lesser welfare significance to the animal than various other afflictions, or possibly due to lack of confidence in how best to rectify lorinery deficits when learning resources or in-country expertise on this topic are scarce and not readily available to practitioners in low- and middle-income countries. Whilst not exploring lorinery specifically, a Brooke-supported survey of 124 working equid welfare practitioners in 12 countries revealed low confidence in harnessing and related topics, with respondents indicating least confidence in fitting body and head harnessing (32% and 29% not confident, respectively) [98].

The design of the bit itself is only one of several potential contributing factors to bit-related welfare impairment. Other pertinent considerations include the manner of driving, the method of attachment of the bit onto the bridle, configuration of the bridle (particularly the nose-band), bit cleanliness and tack maintenance; along with animal-related factors such as dentition, pre-existing oral abnormalities or ingrained behavioural responses (e.g., resistance behaviours whilst in bridle); and the handlers’ skill in using a bridle and bit correctly, expertise in ‘horsemanship’, behavioural recognition and understanding of learning theory [66,99,100,101]. In light of the scope and field-based nature of the current project, it was challenging to control for all of these potentially confounding variables. For animal welfare reasons, drivers were observed when fitting the bit onto the bridle to ensure this was correct and were reminded of the importance of humane driving. As they were already experienced drivers, no particular changes were made to driving practice or any other features of the tack, husbandry regime or vehicle across the study period. Whilst acknowledging the limitation of being unable to carefully control all potential confounding variables, this study does appear to suggest the bit itself as an important contributor to oral injury and resistance behaviour within this cohort of working cart horses. Further study to control for any unintended changes in driving practice, and attempts to ‘blind’ the drivers and observers to the bit type may help to confirm and verify these preliminary findings.

As equid welfare was paramount, this took precedence over the greater robustness and potential for broader extrapolation of results that might have been gained by a random sample. Secondly, as this was field-based research involving working people and horses as opposed to a controlled experimental environment, recruiting a truly random sample would not have been feasible in such locations where the prerequisite data about human and equid populations were not available or reliable. As a charity conducting research on a limited budget, prudent spend of funds on research was also an important consideration influencing scale and sampling methods. The authors considered that adopting a cohort design with a fixed group of willing participants, enabling monitoring, reassessment and subsequent follow up, was the approach most likely to yield findings of practical benefit for the work of Brooke and other working equid welfare practitioners and organisations. However, it is acknowledged that on account of the sampling approach and potential for voluntary response bias, assumptions should not be made that these results can be extrapolated to the total population of drivers and cart horses, as it is possible that there are some demographic differences between the horses of drivers who were willing to participate in the study and were already known to the Brooke team, and those who were not included. Accordingly, further study to validate the findings in a random sample of drivers and horses could be valuable, provided this could be conducted ethically in a way that mitigated potential animal welfare risk.

The sample group was limited to horses working in cart transportation only, because these animals work on a daily basis which enabled evaluation of the quality of the bits in a reasonably short time period, and had also been observed by field staff to be commonly affected by bit-related oral trauma. Furthermore, horses engaged in other work types, such as those used for agriculture, do not work consistently during the dry season. Therefore, it should not be presumed that these results can be extrapolated to working donkeys or mules, nor to equids engaged in different work types, and additional research would be required to ascertain whether similar findings would be emergent for these different equid demographics. As there can be differences in harnessing, bridles and bitting between different working equid groups, it would be advisable to establish the welfare issues specific to the work type, species or working context to enable tailored research into the prevalence, nature and means of amelioration of bit-related welfare issues amongst different working equid demographics. For example, bitting might be informed by differing requirements that drivers encounter in their specific working scenario, and drivers may have different motivations for using bits or bridle types as befits the nature of their work, their level of knowledge and experience, pre-existing cultural norms, or perceptions towards working equids [102]. Exploration of animal welfare issues in conjunction with insights from local equid-owning communities—in this case drivers—is important to devise and implement effective welfare-improvement interventions [45,103,104].

As the project commenced during the COVID-19 pandemic, it was necessary to comply with government-imposed sanitary measures including travel bans between regions in Senegal. That induced delays, but the Brooke team managed to perform many activities remotely or by using the established network of local community-based partner organisations to assist with logistics and participant liaison to enable successful data collection and monitoring. COVID-19-related restrictions did not impact participation in the study by the sample of drivers.

Despite this being a preliminary study of its kind focussed on the specific harness issue of bitting amongst this working equid population, these findings provided confidence to Brooke that a) no unintended negative welfare impacts on the horses had emerged; and b) there would be value in scaling up this project activity to encourage uptake of the new bit designs for cart horses involved in transportation of people and goods, in locations beyond those involved in the current study. As harness-related welfare issues often constitute a ‘hard win’ scenario in working equid contexts, an issue in which improvement can be particularly challenging or marginal [105], these findings are a step towards finding a solution to the problem of bit-related working equid welfare impairment. They also demonstrate the value in adopting an iterative process of design, development, testing in situ and evaluating, conducted in collaboration with local artisans and with the voluntary participation of working cart drivers.

The study yielded additional learning points from the process that may be beneficial for other equid welfare practitioners to consider.

(a) The first challenge was to find local artisans who were sufficiently skilled to design the bits according to the technical specifications provided. In this case, proactively engaging with blacksmiths, who in general have greater experience of metalwork than other groups of artisans, was a successful approach.
(b) The importance of having lorinery knowledge for development of the prototype was recognised. Taking the necessary time to get the prototype right at the beginning, and carefully managing the risk of rolling out an unsuitable product were essential. Artisans, whilst having the metalwork skills, did not have any knowledge of lorinery, and the provision of detailed guidance via an iterative process was fundamental in ensuring the bit design that was ultimately selected was suitable to be taken forward into the trial. It is not recommended to attempt an intervention activity of this type without involvement of a practitioner with specific understanding of equine science, equitation and harness.
(c) In activities of this type, it is not guaranteed that the members of the target demographic will always adopt a new prototype, or that they will continue to use it over the longer term. Accordingly, it is recommended that bit- or harness-improvement activities are coupled with engagement with the intended users, to raise awareness about the benefits for animal welfare and potentially the equid’s productivity, so that there is motivation and willingness to pay a slightly increased cost on account of the benefits.

With regard to future work building on the current study, within Brooke it has been identified that there would be value in attempting to track the horses and drivers involved in this study over several more months in order to assess the bit prototypes over the longer term (e.g., the long-term animal welfare impacts beyond those measured in the current study, such as whether body condition improves as a result of less oral pain and fewer oral lesions). There would be value in assessing other aspects of the project beyond animal welfare (e.g., acceptability of the alternative bits to drivers and equid-owning communities, such as longer-term impacts on the level of control of horses during work, or demand for the new bits sought from vendors). It is also anticipated that the ongoing project will require additional sensitisation activities to help drivers overcome the price differential between the old and new bits, as the former cost 850 CFA (equating to approximately $1.3 USD) and the latter 1250 CFA (equating to approximately $1.9 USD). Planned activities include incorporating education about harmful bits and preferable alternatives in community engagement activities with equid owners and users, demonstrations of the new bit prototypes, educational sessions for drivers, and creation of pictorial booklets and learning materials designed to engage local people (particularly those who are illiterate) with this topic. However, it should be noted that initial uptake of the new bits was very encouraging, as all of the 1000 prototypes produced were distributed, and the demand from drivers exceeded the availability at that time. To address this demand, following the successful trialling of the new bits and completion of the experimental phase, a workshop was arranged for the artisan who had been selected from the initial tender process to train other artisans in each area in production of the successful bit design. The intended outcome is that there is an artisan capable of manufacturing the improved bits in each of the five locations involved in this study, thus making this widely available to several key populations of cart horse drivers, with the ultimate aim of encouraging all drivers using the pre-existing injurious bits to switch to the new designs.

The researchers involved were surprised about the speed at which positive changes in the animal-based indicators were evident during the initial testing phase, with statistically significant differences in behavioural and physical parameters subsequently emergent in the experimental phase over the relatively short time period of the study. This indicated, encouragingly, that activities such as this, which were implemented with a relatively low resource input in terms of staffing and finance, can have a meaningful welfare impact for working horses on a daily basis, and potentially over the longer term throughout their working lives. Furthermore, as there is possibility that the new bit prototypes will persist within equid-owning communities, there is also scope for the current change in practice and associated welfare benefits to be transferred to future generations of drivers and horses too. The sustainability of welfare-improvement interventions of this type are in contrast to, for example, the poor sustainability of one-off or sporadic service-based activities such as veterinary treatment ‘camps’ that only affect the animals on an ad hoc basis, and create no lasting change within communities—indeed, may even create dependency on external intervention that did not previously exist [58].

There is a scarcity of research on working equid harnessing, including lorinery, thus one of the objectives of publication is to enable others to benefit from the learning that occurred from this study in Senegal, and consider the extent to which similar work might be beneficial to explore in other geographical locations and contexts where working equids might also be suffering impaired welfare due to injurious bitting.

## 5. Conclusions

This study has demonstrated the feasibility of producing locally crafted, low-cost aluminium bits for cart horses which can improve upon bit-related aspects of working equid welfare over a relatively short time period of 21 weeks. From a social perspective, findings illustrated that locally produced bits were acceptable to drivers in terms of retaining the necessary control of their horses during work, and that there was recognition of the welfare benefits by these drivers for their horses, as evidenced by the continued usage of the bits by the drivers who participated in the current study and additional demand from drivers who did not. Additionally, this work shows that a relatively simple and cost-effective intervention, implemented in conjunction with voluntary participation of local stakeholders, has scope to perpetuate and proliferate, potentially impacting positively upon large numbers of working horses in Senegal and beyond, whilst also representing a potential livelihood or income diversification opportunity for local artisans who manufacture the improved bits and vendors who sell them.

## Figures and Tables

**Table 1:** Summary of bit specifications. For each factor, the recommendation given to the artisan, its rationale and the animal welfare objective are indicated. Bit images in this table are included to give an indication of type, rather than demonstrate exact designs used in the present study (drawn by Ruth Jobling).

Factor	Recommendations and Rationale	Animal Welfare Objective
Material	- Iron, plastic and aluminium are available and affordable in the context. - Steel not considered due to prohibitive expense. Option 1: Aluminium. - Softer and lighter than iron. - Must be filed completely smooth with no abrasive surfaces or roughness. Option 2: Iron, surrounded by a sheath of solid plastic. - Plastic sheath should encapsulate full length of mouth-piece to avoid risk of pinching the skin at joint with bit cheek-pieces. - Plastic sheath diameter should be wide enough to rotate freely around the iron. - Plastic surface must be completely smooth with no abrasive surfaces or roughness. Recommendation: Proceed to trial both aluminium bits, and iron bits with plastic sheath.	Comfort in the mouth. - No ‘drag’ over the skin. - Material surface must be smooth. - No pinching of oral tissues.
Shape of mouth-piece	Option 1: Mullen mouth-piece (slightly curved). 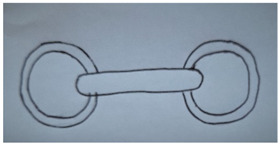 - Gentle curve allows slightly more room for tongue than straight bar; retains fixed shape when pressure is applied with no narrowing (which increases pressure on cheeks) so may improve comfort [60]. - Considered to have milder action than single-jointed mouth-pieces. The latter, when pressure is applied, can alter in shape exerting a ‘nut-cracker’ action with pressure on the cheeks, and greater intra-oral movement [26,36,37]. Option 2: Double-jointed mouth-piece with central lozenge. 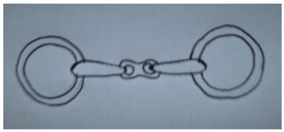 - May relieve bar pressure [61] and avoids opposing action on other side of mouth as occurs with straight or mullen mouth-piece. - However, considered too complicated to manufacture correctly in context; risk of not being made to correct exact specifications (e.g., central lozenge being sufficiently short to avoid bit joints being positioned over bars [26]), or risk of being simplified in future to single-jointed model. Recommendation: Proceed to trial mullen mouth-piece only.	Comfort in the mouth. Gentle action. Mitigation of harm, even when used by ‘heavy-handed’ drivers.
Diameter of mouth-piece	Approximately 1.5 cm diameter. - Narrower mouth-pieces increase the concentration of pressure on a smaller surface area [31,47,59]. - Mouth-pieces that are too thick inhibit equid’s ability to close mouth and jaws comfortably around the diastema (see Figure 7) [37]; can cause similar or greater discomfort than a thin bit [62]. - Horses can typically accommodate a bit of 1.4 cm diameter without incurring tongue compression [37].	Comfort in the mouth. Gentle action. Mitigation of harm, even when used by ‘heavy-handed’ drivers.
Length of mouth-piece	Approximately 0.5 cm of bit on each side of the horse’s mouth when the bit is fitted correctly. - Short mouth-pieces may pinch the lips and pressurise the cheeks against the premolars and molars [36,37,59]. - Long mouth-pieces may have increased lateral movement through the mouth when pressure is applied unevenly on the reins, displacing the bit position and increasing abrasion [37,47].	Comfort in the mouth. Mitigation of harm, even when used by ‘heavy-handed’ drivers. Clarity of signal to animal.
Bit cheek-pieces	Option 1: D-ring cheek-pieces. 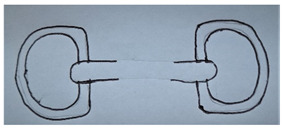 - Reduce risk of bit being pulled through mouth as compared to narrower cheek-pieces [60]. - Minimal scope for poll pressure, due to absence of shank and curb effect [32]. - May help mouth-piece remain in correct position; not susceptible to tilting. - May aid clarity of signalling to turning, as pressure applied along length of D on opposing side. - Coupled with mullen mouth-piece, may encourage correct fitting of bit by users (e.g., cannot be erroneously fitted upside down). Option 2: Full cheek-pieces. 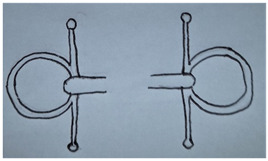 - Possible benefits as for D-ring above [60]. - However, considered at high risk of becoming caught or entangled during the rigours of work, so avoided. Option 3: Half cheek-pieces (as for full cheek piece but with a lower extension only). 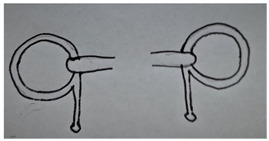 - Possible benefits as for D-ring above [60]. - Lower risk of becoming caught than full cheek-pieces as only has lower extension. Recommendation: Proceed to trial D-ring cheek-pieces and half cheek-pieces.	Comfort in the mouth. Mitigation of harm, even when used by ‘heavy-handed’ drivers. Clarity of signal to animal. Ability to turn.

**Table 2:** Animal-based indicators for monitoring effects of new bit prototypes.

Category	Animal-Based Indicator	Description of Scores
Behavioural (static phase)	Lick/chew	Score 0 = no licking and chewing motions. Score 1 = licking and chewing motions (in absence of food in mouth).
	Facial tension	Score 0 = no tension visible in muscles of jaws, lips or elsewhere on face. Score 1 = tension visible in muscles of jaws, lips or elsewhere on face; muscles may appear more prominent; ears may be back.
Behavioural (locomotion phase)	Open mouth	Score 0 = mouth closed or slightly open; space not clearly visible between upper and lower incisors. Score 1 = mouth open, with lips and teeth separated sufficiently widely that space is clearly visible between upper and lower incisors.
	Tongue loll	Score 0 = tongue not visible. Score 1 = tongue visible protruding or hanging out either side or front of mouth.
	Head toss/shake	Score 0 = head position consistent, or with minimal unintentional movements associated with locomotion. Score 1 = head intentionally moved in sudden up and down motion (toss); and/or rapidly rotated (shake).
	Head tilt/turn	Score 0 = nose in vertical alignment with ears; and neck remains straight and aligned with body orientation. Score 1 = nose not in vertical alignment with ears, as head tilted towards left or right (tilt); and/or neck bent to left or right, out of alignment with body orientation whilst equid is moving forwards (turn) in the absence of signal to turn from driver.
Physical	Lip commissures	Score 0 = no lesions, scabs or bruising. Score 1 = lesion, scab or bruising.
	Tongue	Score 0 = no lesions, scabs or bruising. Score 1 = lesion, scab or bruising.
	Buccal mucosa	Score 0 = no lesions, scabs or bruising. Score 1 = lesion, scab or bruising.
	Bars	Score 0 = no lesions, scabs or bruising. Score 1 = lesion, scab or bruising.

**Table 3:** Demographic information of owners and horses during the testing phase (*n* = 30).

Parameter	Response Category	Number	Frequency
Age group of driver	15–30 years	6	20%
	31–40 years	13	43%
	More than 40 years	11	37%
Driving experience	Less than 1 year	4	13%
	1–5 years	20	67%
	More than 5 years	6	20%
Work type	Transport of people by cart	20	67%
	Transport of good by cart	9	30%
	Undetermined/mixed	1	3%
Age group of horse	Less than 3 years	5	17%
	3–12 years	14	47%
	More than 12 years	11	36%
Sex of horse	Stallion	30	100%
	Mare	0	0%

NB: All horses were male, as mares are not used for commercial activities. Geldings were not observed as castration is rare in the context.

**Table 4:** Physical and behavioural indicator prevalence at beginning and end of testing phase (*n* = 30).

	Indicator	Count at Beginning of Testing Phase	Count on Completion of Testing Phase
Physical indicators (lesions)	Lip commissures	28	19
	Tongue	1	0
	Buccal mucosa	8	0
	Bars	1	0
Behavioural indicators (static phase) – pre-existing bit	Lick/chew	16	N/A
	Facial tension	2	N/A
Behavioural indicators (static phase)—new bit	Lick/chew	10	0
	Facial tension	0	0
Behavioural indicators (locomotion phase)	Open mouth	5	0
	Tongue loll	2	0
	Head toss/shake	2	0
	Head tilt/turn	2	0

**Table 5:** Demographic information on drivers and horses during the experimental phase (*n* = 540).

Parameter	Response Category	Number	Frequency
Age group of driver	15–30 years	86	16%
	31–40 years	362	67%
	More than 40 years	92	17%
Driving experience	Less than 1 year	0	0%
	1–5 years	232	43%
	More than 5 years	308	57%
Work type	Transport of people by cart	329	61%
	Transport of goods by cart	211	39%
Age group of horse	Less than 3 years	16	3%
	3–12 years	481	89%
	More than 12 years	43	8%
Sex of horse	Stallion	540	100%
	Mare	0	0%

## Data Availability

The data presented in this study are available on request from the corresponding author.
